# Experiences of nurses on COVID-19 preventive protocols implementation in Tamale Metropolis, Ghana: A qualitative exploration

**DOI:** 10.1371/journal.pgph.0001674

**Published:** 2023-06-26

**Authors:** Martin Nyaaba Adokiya, David Abatanie Kanligi, Michael Boah

**Affiliations:** 1 Department of Epidemiology, Biostatistics and Disease Control, School of Public Health, University for Development Studies, Tamale, Ghana; 2 Department of Social and Behavioral Change, School of Public Health, University for Development Studies, Tamale, Ghana; 3 Pediatric Unit, Savelugu Municipal Hospital, Ghana Health Service, Northern Region, Ghana; PLOS: Public Library of Science, UNITED STATES

## Abstract

The Coronavirus Disease, 2019 (COVID-19) disrupted healthcare delivery. Health workers, particularly nurses are key members of the interdisciplinary healthcare team. They are faced with many challenges due to the pandemic. In addition to providing basic healthcare services, nurses are required to adhere to the COVID-19 recommended safety protocols. This study explored experiences of nurses on the implementation of COVID-19 preventive protocols in Tamale Metropolis, Ghana. A qualitative study was conducted among seventeen (17) nurses, comprising five (5) staff with COVID-19 infection, and twelve (12) ward managers or in-charges who did not have COVID-19 infection, using explorative design and an interview guide. The participants were purposively selected. The ward managers/in-charges and infected staff were interviewed face-to-face and by mobile phone respectively. Content analysis was conducted on the data and the results presented as themes and sub-themes. After the analysis, five (5) main themes and fourteen (14) sub-themes were identified on experiences of nurses regarding COVID-19 preventive protocols implementation. These included understanding COVID-19 transmission/spread (patients-to-staff, staff-to-staff and through fomites), communicating the preventive protocols (social media, ward meetings and administrative memoranda), and attitude of nurses on the protocol’s implementation (growing apathy, discomfort in applying personal protective equipment (PPEs) and outright defiance). Nurses also experienced some challenges and inadequate support (progressive decline in supply of PPEs, infrequent supply of water and limited infrastructure), in addition to dealing with issues of protocols implementation in healthcare setting (inapplicability of social distancing in hospital setting and improvising PPEs). In conclusion, the nurses had varied experiences on COVID-19 preventive protocols implementation. The themes explored were mode of COVID-19 transmission, communication approaches, negative attitudes, inadequate logistics and inability to implement social distance. Overall, these affected the effective implementation of the protocols. Thus, health facilities should be provided with adequate logistics/supplies and trainings to enable nurses implement COVID-19 preventive protocols effectively.

## Introduction

Coronavirus Disease of 2019 (COVID-19) emerged in 2019. It spread widely and was declared a pandemic on 11^th^ March, 2020 by the World Health Organization (WHO) [1]. The impact of the pandemic has been enormous as it affected social, economic and political aspect of countries, with the health sector disproportionately impacted globally [2–4]. Health care workers have been the frontline fighters of the pandemic. They provided care at the peril of their lives through frequent exposures. However, inadequate logistics in the form of personal protective equipment (PPEs), limited human resources, lack of training, and increased worked load, have exposed most health workers to physical and psychological trauma [5]. The safety of clinical staff such as nurses is crucial to controlling COVID-19. Safety measures seek to limit spread of the virus within the healthcare settings. Consequently, evolving understanding of COVID-19 transmission led to postulation of safety protocols including patients isolation, physical distancing, hand hygiene practices and wearing of PPEs [6]. However, logistic shortfalls disrupted effective implementation of the preventive protocols. The situation is particularly pronounced in developing countries’ setting where basic PPEs kits and institutional support is generally lacking, making response to the pandemic more challenging [7].

Nurses are key members of the interdisciplinary healthcare team and they play pivotal role in the COVID-19 pandemic response [8,9]. With limited resources, nurses are exposed whilst caring for patients [10]. As a result, more nurses contracted COVID-19 than any other category of health workers with an estimated 38.6% of all health worker infections globally [11]. In Ghana, nurses and doctors constitute the majority of the over 2000 infected health workers reported [12,13]. Similarly, 37.5% of health worker infections in the Northern Region of Ghana were contributed by nurses [14]. Increasing exposure and infection of nurses jeopardize patients’ safety. Thus, exploring nurses’ experiences on COVID-19 safety protocols implementation would provide policy makers with additional insights on the preventive strategies. Hence, this study explored the experiences of nurse on the COVID-19 preventive protocols implementation at health facilities in the Tamale Metropolis of northern Ghana.

## Materials and methods

### Study design

This study employed a qualitative approach, with an explorative design to collect data from nurses, using a semi-structured questionnaire. Face-to-face or in-depth telephone interviews were conducted among nurse managers and nurses with history of COVID-19 infection from 19^th^ January to 26^th^ February 2021. Data analysis followed a qualitative content analytic approach as proposed in previous studies [15,16]. The results were presented as themes and sub-themes.

### Study setting and participants

The study was conducted among twelve (12) nurse mangers (ward in-charges) and five (5) nurses with history of COVID-19 infection. The participants were purposefully selected from six public health facilities that form the main health delivery system of the Tamale Metropolis of Ghana [17]. The facilities included three hospitals (Tamale Teaching Hospital, Tamale Central Hospital and Tamale West Hospital) and three health centers (Nyohini, Moshie-Zongo and Vitting Health Centers). Inclusion criteria was based on participants’ role as nurse supervisor (being a ward in-charge/assistant ward in-charge) and having history of COVID-19 infection and willingness to participate in the study. A total of seventeen (17) nurses participated in the study (Table 1).

**Table 1:** Sociodemographic characteristics of study participants (n = 17).

S/N	Gender	Age (years)	Description/Role	Work experience (years)
1	Female	62	Ward In-Charge	32
2	Female	45	Ward In-Charge	18
3	Male	37	Ward I-n-Charge	11
4	Female	39	Ward In-Charge	17
5	Male	35	Ward In-Charge	7
6	Male	38	Ward In-Charge	5
7	Male	42	Ward In-Charge	13
8	Female	36	Ward In-Charge	10
9	Female	40	Ward In-Charge	10
10	Female	41	Assistant Ward In-Charge	6
11	Female	29	Assistant Ward In-Charge	2
12	Female	32	Assistant Ward In-Charge	3
13	Female	23	Infected Staff	1
14	Male	25	Infected Staff	1
15	Male	31	Infected Staff	3
16	Female	27	Infected Staff	2
17	Male	33	Infected Staff	5

### Data collection procedure

Purposive sampling technique was employed to recruit participants for the study. An interview guide with open ended questions covering the study objective was developed and used to collect the data. The time and/or location for the interviews were determined and agreed upon by the participants. For the purpose of consistency, one researcher (DAK) conducted all the interviews. The findings were then cross-checked with the other members (i.e., MB and MNA) of the team until a consensus was reached.

At the outset of the interviews, we began by asking general questions that pertained to the objective of our study. These included inquiries about the spread of COVID-19 within the health facility, the guidelines in place for COVID-19 prevention, and the opinions of the ward in-charges regarding these guidelines. For those staff members who had previously contracted COVID-19, we asked questions about their compliance with preventive protocols prior to infection, and how they perceived their work conditions to have influenced their infection status. Subsequently, we posed more specific and in-depth questions, such as how social distancing was maintained during patient care, the challenges encountered while adhering to COVID-19 preventive protocols, and measures taken to ensure the timely detection of COVID-19 cases. To gain clarity and obtain further details on participants’ responses, we utilized follow-up questions such as requesting for elaboration, examples, and further explanations of their viewpoints. The interview sessions took between 20 and 45 minutes to complete.

The sampling process was concluded after reaching data saturation, which was determined when no new codes could be generated from subsequent interviews [18]. Data saturation was achieved after the 15^th^ interview. However, in order to further validate and confirm data saturation, two additional interviews were conducted with ward-in-charge participants. With the exception of the five (5) nurses who had a history of COVID-19 and were interviewed via phone calls, all other interviews were conducted in person, face-to-face. Prior consent from the participants was obtained, and all interviews were audio recorded to ensure accuracy and reliability of data collection.

During the data collection process, the researchers adhered to all COVID-19 safety directives to ensure the well-being of both the participants and the research team. This included wearing masks, practicing thorough hand hygiene, and maintaining physical distancing measures as recommended by relevant health authorities.

### Data analysis

Content analysis was conducted on the data. The analysis followed similar approach proposed by Graneheim and Lundman [19]. The audio records of the interviews were listened to severally and transcribed verbatim. The transcripts were read several times to gain a general concept of the information. The texts were then read word-by word and with great care, taking into consideration key words that form initial ‘meaning units’. The meaning units were condensed into generalized ideas and further summarized into codes, which reflected the initial meaning units. We then merged and categorized the codes depending on similarity of meaning/concept and association with each other. The categories were compared with each other and sorted into main and sub categories. This abstraction led to the formation of main themes and sub-themes (Table 2). The level of abstracting and theme formation is attached as supplemental material (S1 Table). The entire analysis was discussed and agreed upon by all authors.

**Table 2:** Main themes and sub-themes derived from study participants (n = 17).

Theme	Sub-theme
1. Understanding COVID-19 spread or transmission	From patients-to-staff
	From staff-to-staff
	Through fomites
2. Communicating preventive protocols	Social media
	Administrative memos
	Ward meetings
3. Nurses attitude in lieu of protocols implementation	Growing apathy
	Discomfort in applying PPEs
	Outright defiance/disregard
4. Dealing with challenges and issues of inadequate support	Progressive decline in supply of PPEs
	Infrequent supply of water
	Limited infrastructure
5. Protocols implementation in healthcare	Social distancing in health care setting
	Improvising PPEs

### Rigor of findings

Rigor of this study was attained by adopting Lincoln and Guba’s criteria: credibility, dependability, confirmability, transferability and authenticity of findings [20]. The researchers ensured prolong engagement with data, member checking and peer debriefing to ensure credibility of findings. For member checking, the final codes were presented to three participants to confirm that their viewpoints were captured. The researchers also invited three experts to validate the codes and data analysis process as a way of ensuring peer checking. Dependability of the study result was achieved by comprehensively describing the methods of coding and presenting thick and rich text of participants’ experiences. To ensure the confirmability of information, codes drawn from each interview were sent back to participants for verification. When necessary, corrections were made to the initial codes to reflect participants’ perspectives. The selection and characteristics of the participants, methods of data collection, analysis and documentation of participants’ quotes were based on the study objective and comprehensively described to enhance transferability of the study findings.

### Ethics consideration

This study was approved by the Kwame Nkrumah University of Science and Technology (KNUST) Ethics Board and the Ghana Health Service Ethics Review Committee with review numbers CHRPE/AP/493/20 and GHS-ERC 026/02/21 respectively. Prior to the interviews, the study objective was explained to each participant. The participants decided on the place and /or time for the interviews to be conducted. Each participant signed a written informed consent form and were assured they could withdraw from the study at any point without consequences. The participants were also assured of anonymity and confidentiality of information they provided.

## Results

A total of seventeen (17) interviews were conducted among twelve (12) ward in-charges and five (5) staff with history of COVID-19 infection. In all, ten (10) of the participants were females. The ward in-charges included four (4) males and eight (8) females. They had an average work experience of 10.6 years (ranging from 2 to 32 years). The five (5) staff with history of COVID-19 infection included three (3) males and two (2) females. Their years of experience ranged from 1 to 5 years, with an average of 2.4 years (Table 1).

### Themes and sub-themes

Exploring nurses’ experiences on COVID-19 preventive protocols implementation resulted in five (5) main and fourteen (14) sub-themes. The main themes of the study included; understanding COVID-19 spread, communicating preventive protocols, nurses’ attitude in lieu of protocols implementation, dealing with challenges and issues of inadequate support, and protocols implementation in healthcare (Table 2). The level of abstraction of themes and sub-themes is outlined in S1 Table.

### Understanding COVID-19 spread/transmission

The participants provided responses to address their understanding of COVID-19 spread or transmission. According to the participants, COVID-19 is spread in diverse ways. They also reported that understanding the mechanism of spread of the virus is crucial for taking preventive measures. In addition to the main theme, three sub-themes were identified representing the route of spread/transmission including from patients-to-staff, from staff-to-staff and through fomites.

### From patients to staff

From the participants’ experiences, COVID-19 was mostly spread at the health facility by infected patients. They described COVID-19 patients as the primary source of infection in the health facilities. The participants further elaborated that most staff who contracted COVID-19 might have been infected by patients they encountered during work. From the account of the ward in-charges, the problem arose because some patients were admitted into the wards before they were diagnosed of COVID-19.

‘‘*COVID-19 is spread at the facility by infected patients*, *particularly the undiagnosed ones*. *This happens when the patient coughs*, *sneezes or talks*. *We have had three staff who got infected in this ward by patients*. *COVID-19 mostly begins with general symptoms*, *so by the time the patient is diagnosed*, *he/she would already have a long list of contacts*, *some of whom would surely contract the virus as it happened in our ward” (***P6, ward in-charge***)*.

A staff with history of COVID-19 infection alluded to the ‘patient-to-staff’ mode of transmission. The staff related the personal infection status to exposure to an undiagnosed COVID-19 patient they had tried resuscitating.

‘‘*Exposure to a suspected patient is the main cause of the spread*. *I think I got infected by one patient we attempted resuscitating*. *I did not know he was a suspected case*. *I joined in the team and later found that the patient had COVID-19*. *Five days later*, *I lost my sense of smell*. *It was when I realized I was infected”* **(P15, infected staff)**.

### From staff-to-staff

Insights from the interviews indicated that staff who contracted COVID-19 also posed risk to their colleagues. Accordingly, some staff with history of COVID-19 transmitted the virus to their colleagues.

‘‘*Yeah*! *For me*, *staff-to-staff spread was also a source*. *We have seen that in our facility too*. *We recorded an incident of staff-to-staff transmission here*. *One of us was infected and they tested all the staff she came in contact with*. *Two other results came out positive”* **(P10, ward in-charge***)*.

### Through fomites

Some of the participants were of the view that transmission of the virus also occurred via inanimate objects. They indicated that activities of COVID-19 patients spread the virus in the environment which became a source of infection.

‘‘*The virus is also spread on surfaces*, *and when one touches it*, *he/she can transfer it to the eyes*, *mouth or nose*. *When the patient coughs*, *sneezes or talks*, *they spread the virus around their surroundings*. *In this way*, *both the patient and his/her immediate environment become source of infection…” (***P6, ward in-charge***)*.

#### Communicating preventive protocols

On how COVID-19 preventive protocols were communicated to staff working in the health facilities, it was reported that the ward in-charges used various channels to communicate protocol information. The common communication channels identified were social media, ward meetings and administrative memoranda.

### Social media

According to the participants, social media was the main channel of information delivery when the pandemic emerged. They reiterated that measures including restrictions, staff duty shift implemented against COVID-19 spread made it difficult for all the staff to meet regularly. However, they needed to keep members abreast with regular updates. Consequently, social media platforms came in handy. The participants frequently cited WhatsApp as the most utilized conduit for disseminating COVID-19 related information.

‘‘*Oh*! *COVID-19 is talk of the day*. *Every day we talk about it*. *The main channel of communication is our WhatsApp platform*. *We hardly meet these days because of the change in our shift system*. *We have divided ourselves into two groups that alternate weekly*. *These groups hardly meet*. *But the platform gives us the opportunity to still interact*, *and update ourselves on current happenings on the pandemic” (***P5, ward in-charge***)*.

“*Yes*! *On WhatsApp*, *on daily basis*. *Because we are at risk and I need to communicate to my staff*. *Any time there is development*, *I communicate with them*. *I just communicated about it* [COVID-19] *to my staff using the WhatsApp” (***P1, ward in-charge***)*.

### Ward meetings

Though COVID-19 limited physical contacts, the participants reported having ward meetings. This is because the ward meetings were also a medium for delivering COVID-19 related updates to nurses. According to the participants, the staff met on few occasions to discuss issues relating to the pandemic. In addition, staff shared information during handing and taking over sessions of the ward.

“*We met at the end of each shift and briefed ourselves of happenings in the ward*. *We discussed the state of the ward before handing/taking over*. *During these meetings*, *in-coming staff are informed of developing issues including updates on suspected cases and new management protocols that might have emerged*. *I think information about the pandemic is not hidden” (***P2, ward in-charge).**

### Administrative memoranda

Administrative memoranda were also utilized to communicate COVID-19 updates to staff. The ward in-charges recounted that they regularly received updates via memoranda which they post at vantage points for staff to read. Thus, the staff got informed passively on updates about the pandemic through administrative memoranda.

“*We get memos* (memoranda) *on the pandemic from administration at all times*. *I post them at vantage points including our notice board and even on the walls in the ward*. *You can see some here [*pointing to the wall*]*. *The memos are really helpful*. *They help us get current updates on the pandemic and its management”* (**P8, ward in-charge**).

#### Nurses’ attitudes in lieu of protocols implementation

The responses of the participants led to the development of the third theme. It relates to issues regarding nurses’ attitude on COVID-19 preventive protocols implementation. This theme comprises three sub-themes namely; growing apathy, discomfort in applying PPEs and outright disregard of preventive protocols.

### Growing apathy

Based on the participants’ experience, nurses’ enthusiasm regarding implementation of COVID-19 preventive protocols began to wane with time. They asserted that nurses were more concerned with COVID-19 preventive protocols in the first wave of the pandemic compared with subsequent episodes.

‘‘*People used to be so concerned about COVID-19*. *But it’s not the same any longer*. *It’s not because they don’t know*, *but people care less about the preventive measures*. *For instance*, *there were staff stationed at the entry points* [of the facility], *checking people’s temperature and assessing for possible symptoms*. *But now it’s not there*. *So*, *I think there is a general apathy to COVID-19*, *not only within the public domain*, *but among nurses as well”* (**P1, ward in-charge**).

‘‘*Hmmmm*, *there seem to be some level of apathy towards the pandemic*. *My staff used to be serious*, *but for now they don’t care much*. *Some will even walk in without face mask*, *and when you ask*, *they will be like they have forgotten*. *It’s unlike during the first wave when everyone was serious about the protocols”* (**P10, ward in-charge**).

### Discomfort in applying Personal Protective Equipment (PPEs)

The participants reported that among the nursing staff, the use of PPEs can sometimes be discomforting. As a result, they deliberately breached COVID-19 preventive protocols on the grounds of personal conform. A staff with history of COVID-19 infection tried to explain why she was not always wearing face mask.

‘‘*I was wearing the mask*. *But I don’t often feel very comfortable*. *So*, *I used to take it off once awhile to take fresh air [*giggled*]*. *Besides*, *it’s difficult breathing through it*. *So*, *when I’m not interacting with patients*, *I sometimes take it off for a while*. *You know*, *the work was overwhelming and I usually get very exhausted*, *and it’s like wearing a face mask even made it worst”* (**P16, infected staff**).

### Outright defiance

Some of the participants reported that they also experienced outright defiance to the implementation of COVID-19 preventive protocols by health staff. Accordingly, the staff would intentionally breach the protocols for no reason. The ward in-charges reported that though the staff were aware of the directives on the implementation of the preventive protocols in the health facilities, they deliberately chose to disregard them with the consequences of exposure and infection.

‘‘*Some of my staff do not want to adhere to the COVID-19 safety protocols*. *I have complained severally*, *and even confronted some of them about it*. *It is not just that they do not know about the protocols*, *but mostly they deliberately would not comply… I just have to keep talking to them*” *(***P12, ward in-charge***)*.

In view of these deliberate breaching of the protocols, some ward in-charges had to issue punitive measures to check poor attitudes of their staff:

*‘‘We have staff who do not want to comply with the protocols*. *They know but would not comply*… .*We have had to use queries as a strategy to enforcement compliance*. *I issued queries to some of my staffs who knew of the protocols but failed to comply” (***P3, ward in-charge***)*.

“*Yes*, *some staff have behavioral problems*. *Some staffs would not comply no matter what you do*. *Sometimes you have no option than take disciplinary actions*. *There were few instances I have to sit some staff down and admonish them because of their poor attitude to the safety protocols” (***P7, ward in-charge).**

#### Dealing with challenges including issues of inadequate support

The fourth theme of the study focuses on challenges and issues of inadequate support for the implementation of the COVID-19 preventive protocols. There are three sub-themes including progressive decline in supply of PPEs, infrequent supply of water and limited infrastructure.

### Progressive decline in supply of PPEs

The participants experienced gradual reduction in the supply of personal protective equipment for the prevention, control and management of COVID-19 pandemic. We found that most organizational support dwindled after the first wave of the virus.

‘‘*Supply of PPEs was okay*. *As for last year*, *we had adequate supplies [*PPEs*]*. *Every week we used to get supplies*. *But about a month ago*, *we started having shortages*. *For now*, *there is severe shortage of PPEs*, *especially gloves and face mask”* (**P1, ward in-charge**).

According to some of the ward in-charges, it became necessary to improvise PPEs as a coping mechanism. They explained that staff were compelled to reuse some ‘single-use’ PPEs after they failed to get supplies from the health facility stores.

‘‘*Hmmmmm…[*sighed*]…*.*as for that one*, *it’s a problem*. *Lately*, *we have serious shortages*. *But it was not like that*. *We used to be given hand sanitizers*, *gloves*, *and face masks in adequate quantities during the first wave*. *For now*, *it is difficult for us*. *We are just improvising*. *We have resorted to reusing some single use PPEs…”* (**P2, ward in-charge**).

‘‘*Logistics*, *we have had short fall in logistics*. *Supply from the stores has not been coming regularly*. *Unlike during the first wave when we even used to get donations*, *currently we have severe shortages”* (**P3, ward in-charge**).

These experiences were corroborated by that of some of the staff who got infected with COVID-19. These infected staff confirmed the challenges encountered by the other participants of this study. Accordingly, the staff had to resort to buying their own PPEs for personal use.

‘‘*The facility should have ensured we were given adequate protection*. *We were barely given enough PPEs*. *There were times patients will come and you have no gloves to work with*. *It was really frustrating*. *As for things like face mask*, *you do not need to be told to buy your own*. *Once a while*, *they would give us cloth mask (*locally manufactured mask from a cloth material*)” (***P14, infected staff***)*.

*‘‘I think I got infected because we did not get adequate protection*. *Being at the forefront*, *having first time encounter with patients coming into the facility and not having enough PPEs was not cool at all*. *In fact*, *it was scary at the time and I sometimes contemplated quitting nursing*. *It is like some of us just exposed ourselves for nothing” (***P13, infected staff***)*.

### Infrequent water supply

The participants also experienced water supply challenges. They reported that though handwashing was mandatory, there were times they run out of water at the health facility. consequently, the participants had to rely on other alternatives like hand sanitizer for hand hygiene while providing health care at the facility.

‘‘*…The tap was not flowing frequently*. *Sometimes*, *you can go through a whole shift without the tap flowing*. *So*, *we were depending on sanitizers for hand hygiene*. *The situation was just that bad”* (**P17, infected staff**).

### Limited infrastructure

The participants also encountered challenges on available infrastructure for COVID-19. The participants bemoaned the small size of the wards at the health facilities which made it difficult for patients spacing. In addition, there were inadequate isolation rooms to admit suspected cases. As a result, social distancing in patient care could not be effectively implemented.

‘‘*…errrrhm* [stammered*]*, *the ward is too small*. *It is difficult to space out patients here*. *Even when there is a suspected case*, *it becomes difficult to quarantine*. *It is not every patient with respiratory symptoms that must be sent to the isolation unit*. *You see*, *it is not morally right to admit a patient to isolation without first testing*. *Respiratory symptoms could point to any other condition than COVID-19” (***P6, ward in-charge***)*.

#### Implementing the protocols in healthcare

After the analysis, we also created a theme on implementing COVID-19 preventive protocols in health facilities. In addition, two sub-themes namely; social distancing in healthcare setting and improvising PPEs were created based on the responses of the participants.

### Social distancing in healthcare setting

From the participants’ experiences, it was not possible to implement all the COVID-19 preventive protocols at the health facilities. They reported that social distancing protocol could not be applied rigorously in healthcare within the setting. Accordingly, the staff had to adopt alternative measures.

‘‘*My son*, *where have you heard that*? *Social distancing is impossible during patient care*. *We have to keep to the safety protocols; wearing of the face mask*, *frequent hand washing and sanitizing hands” (*P1**, ward in-charge***)*.

‘‘*As you are aware*, *there is no distancing between nurses and patients*. *Most of the patients here are high dependent cases*, *and you have to help them do everything*. *Feeding*, *lifting*, *and bathing them are our responsibilities*. *So*, *we cannot actually socially distance…” (***P9, ward in-charge***)*.

Regardless of these challenges, some participants revealed that they used different approaches to attain social distancing. The measures they implemented to avoid contracting COVID-19 included social distancing. This was achieved through limiting the number of staff and patient intake to ensure health workers and patients safety during work.

‘‘*There are written guidelines for both staff and patients*. *We have been complying with the protocols*. *We used the shift system to reduce the number of staff in the ward*. *Also*, *we reduce patient intake*. *We wash our hands*, *and sanitize them in-between tasks as well” (***P4, ward in-charge***)*.

### Improvising PPEs

As a result of inadequate PPEs and supplies, the participants reported that personal protective equipment were being improvised. The logistic challenges were consistently cited as the causes of ineffective implementation of COVID-19 preventive protocols. The participants explained that shortfall in supply of logistics limited the use of PPEs during work.

‘‘*Because of the shortages in supply of logistics*, *we are now managing*. *We are improvising and managing*. *We have to prioritize the use of gloves especially*. *Unless it is needful* [stressed] *we don’t wear gloves*. *If it is not necessary really*, *we don’t don gloves*” *(***P3, ward in-charge***)*.

## Discussion

Our study explored the experiences of nurses on COVID-19 preventive protocols implementation in Tamale Metropolis of Northern Ghana. The study identified five main themes. These are understanding COVID-19 spread, communicating preventive protocols, nurses’ attitude in lieu of protocols implementation, challenges including inadequate support, and protocols implementation in healthcare setting.

In the current study, the participants reported three mechanisms of COVID-19 spread namely patient-to-staff, staff-to-staff and through fomites. These mechanism of the virus transmission conform with earlier reports that COVID-19 is transmitted via person-to-person or fomites [21,22]. This finding has implication for communicating COVID-19 preventive measures. Accordingly, developing measures to limit spread of the virus requires an understanding of its mechanism of spread [21]. Therefore, this finding may be suggestive of nursing managers taking appropriate measures for the safety of staff based on their understanding of the pandemic.

Our finding revealed multiple channels of information delivery to the public, particularly, health workers. The participants recounted using social media, administrative memoranda and meetings to disseminate COVID-19 related updates or information. In this study, social media was predominantly used to deliver information to staff due to ease of access. This finding is similar to an earlier report that social media channels were becoming a valuable source of information on the pandemic [23]. Social media helps to maintain continuous flow of information even in instances where human interaction is not possible. Besides, regular risk communication keeps health workers informed and thereby improving their attitude towards COVID-19 preventive measures [24,25]. In contrast, a previous study reported that lack of information and proper understanding contributed to many health workers contracting the virus during the initial stage of the pandemic [26]. This calls for renewed efforts to ensure that health workers including nurses have continuous access to regular updates or information on the pandemic. Consequently, the use of multiple channels for communicating updates should be sustained for effective dissemination of COVID-19 related updates.

Our findings also showed that nurses experienced challenges and inadequate support on COVID-19 preventive protocols implementation. The study participants bemoaned the paucity of PPEs coupled with infrequent water flow. Additionally, increased work load was reported due to the prolonged shift system as well as absentee staff. The inadequate PPEs and increased work demand has been reported in other countries [27,28]. COVID-19 brought unforetold challenges to healthcare delivery with increasing admissions and a corresponding need to utilize more consumables [29]. Logistic inadequacy became pronounced in the peak of the pandemic. Thus, the short falls in supply of logistics is a universal challenge in the fight against COVID-19. Regardless, health workers were mandated to work which made it necessary to improvise PPEs; thus, increasing exposure to the virus. The need to provide adequate support in terms of adequate PPEs, allocation of human resources and training of health workers have been emphasized [25,30]. Therefore, health facilities should develop robust emergency response strategies to counteract the impact of future health emergencies like COVID-19.

We found that nurses had negative attitude regarding COVID-19 preventive protocols implementation. There were reports of apathy, discomfort in applying PPEs and disregard of the preventive protocols. Similar findings have been reported in Venezuela where health workers exhibited negative attitude to COVID-19 prevention [31]. Studies in China also reported that nurses were uncomfortable with full gear PPEs as they experienced overheat, dehydration and headache which impacted their sense of vision, hearing and judgement [32]. The use of PPEs mostly interfered with smooth communication between care providers and patients [33]. With these inconveniences, health workers have good reasons to resist PPEs usage. Resistance to use PPEs may stem from issues relating to personal comfort or adverse effects experienced from prolong usage. Additionally, the use of PPEs is reported to interrupt smooth delivery of care [34,35]. Thus, prolonged use of PPEs may become undesirable to health workers. Hence, appropriate coping strategies should be included as part of the training on PPEs usage for health workers.

This study also explored experiences of participants on COVID-19 preventive protocols implementation in healthcare setting. It was reported that some of the protocols like social distancing could not be implemented effectively. In addition, reduced logistics supply compelled staff to improvised PPEs at their health facilities. Health workers in the southern part of Ghana also reported that social distancing was ineffectively implemented in patient care [36]. The pandemic resulted in a rapid deployment of safety protocols to curtail the virus spread [37]. However, the preventive measures were not meant to be applied universally in all settings. The health care sector has peculiar demands which requires revision of some guidelines. Experiences from previous pandemics show that social distancing protocol may be efficient in slowing viral transmission within communities, but not applicable in healthcare settings [38,39]. However, higher infection of health workers in non-COVID-19 patient wards supports the call to enforce social distancing in every healthcare setting [40]. Thus, health facility management can achieve social distancing by reducing staff numbers, using video conference calls for ward meetings, rearranging staff seating arrangements and reminding health workers to social distance with posters [41]. This corroborates our finding that the number of staff were reduced and scheduled on a weekly shift system to reduce overcrowding in the health facilities. Some authors have argued that social distancing may not be attained with a straightforward approach within the healthcare systems. Thus, achieving social distancing may be theoretical than practically implemented in healthcare settings [41]. It requires adapting various strategies depending on each situation [39]. Therefore, PPEs may fill the gaps when social distancing is not achievable. However, issues of supply shortages confronting the healthcare system made it imperative to improvise usage of PPEs, particularly in the absence of alternatives [42–44]. Consequently, many health workers were anxious, exposed and infected with the virus. This pandemic revealed the unpreparedness of the global community for emergence and reemergence of pandemics. The lessons learnt should serve as a wakeup call for addressing future health emergencies. Emergency response teams should be setup as an integral part of our healthcare systems. These should be decentralized to all healthcare establishment across the country with properly defined roles and supplied with adequate logistics.

## Conclusion

This study explored experience of nurses on COVID-19 preventive protocols implementation in healthcare setting. There were different mechanisms of the virus spread and various communication channels were used to disseminate information on preventive protocols. Additionally, poor attitude of nurses, coupled with inadequate logistics supply impeded effective implementation of the protocols. We suggest that facility managers should provide adequate support including logistics and training to enable nurses implement COVID-19 preventive protocols. In addition, multiple communication channels, development of emergency response strategies and teams need urgent attention.

## Supporting information

S1 Table Level of abstraction of themes and sub-themes. (DOCX)

## Abbreviations

CHRPE: Committee on Human Research Publication Ethics
COVID-19: Coronavirus Disease of 2019
ERC: Ethics Review Committee
GHS: Ghana Health Service
IPC: Infection prevention and control
KNUST: Kwame Nkrumah University of Science and Technology
PPE: Personal protective equipment
WHO: World Health Organization
