# Supplementary material for: Experiences of nurses on COVID-19 preventive protocols implementation in Tamale Metropolis, Ghana: A qualitative exploration
Source: PLOS Glob Public Health. 2023 Jun 26;3(6):e0001674. doi: 10.1371/journal.pgph.0001674 (PMC10292701; doi:10.1371/journal.pgph.0001674)
Supplement: S1 Table — (DOCX) [file pgph.0001674.s001.docx]

**S1 Table: Level of abstraction of themes and sub-themes**

**Overarching theme:** **Experiences of nurses on COVID-19 preventive protocols implementation**

| **Meaning units** | **Condensed meaning units** | **Codes** | **Themes and sub-themes** |
| --- | --- | --- | --- |
| “COVID-19 is spread in the facility by infected patients. They spread the virus to staff, other patients or the hospital environment’’.  ‘‘Exposure to a suspected patient is the main cause of the spread”.  “When the patient coughs, sneezes or talks, they spread the virus around their surroundings which makes, both the patient and his/her immediate environment source of infection”. | Spread of COVID-19 through patients, staff or hospital surrounding (tables, door handles, beds) | Patients.  Staff  Fomites | 1. **Understanding COVID-19 spread or transmission**   From patients-to-staff  From staff-to-staff  Through fomites |
| “Any time there is update, I communicate with my staff using the WhatsApp…”  “We get memos (memoranda) on the pandemic from administration at all times”.  “We meet at the end of each shift and brief ourselves of happenings on COVID-19”. | COVID-19 updates disseminated via WhatsApp, administrative communique and staff interaction | WhatsApp updates  Administrative communiques  Ward interactions | 1. **Communicating preventive protocols**   Social media  Administrative memos  Ward meetings |
| ‘‘People used to be so concerned about COVID-19, but it’s not the same any longer”.  ‘‘I was wearing the mask, but I don’t often feel very comfortable”.  “It is not just that they do not know about the protocols, but mostly they deliberately would not comply”. | No more concern for COVID-19  PPEs are not comfortable  Refusing to comply with COVID-19 protocols | Diminishing concern  PPEs discomfort  No compliance | 1. **Nurses attitude in lieu of protocols implementation**   Growing apathy  Discomfort in applying PPEs  Outright defiance/disregard |
| ‘‘Logistics, we have had short fall in logistics. Supply from the stores has not been coming regularly unlike during the first wave”.  ‘‘…The tap was not flowing frequently. Sometimes, you can go through a whole shift without the tap flowing”.  The ward is too small. It is difficult to space out patients here. Even when there is a suspected case, it becomes difficult to quarantine”. | PPEs supply reduced  Water was not always available  Small nature of wards | Decline in PPEs  Interruption in water flow  Small wards | 1. **Dealing with challenges and issues of inadequate support**   Progressive decline in supply of PPEs  Infrequent supply of water  Limited infrastructure |
| ‘‘As you are aware, there is no distancing between nurses and patients. Most of the patients here are high dependent cases, and you have to help them do everything”.  ‘‘Because of the shortages in supply of logistics, we are improvising and managing. We have to prioritize the use of gloves especially”. | Social distancing between nurses and patients  PPEs shortage led to improvising | Health care social distancing  Improvising | 1. **Protocols implementation in healthcare**   Social distancing in health care setting  Improvising PPEs |
